# Activity Profile and Energy Expenditure Among Active Older Adults, British Columbia, 2011–2012

**DOI:** 10.5888/pcd12.150100

**Published:** 2015-07-16

**Authors:** Kenneth M. Madden, Maureen C. Ashe, Jocelyn M. Chase

**Affiliations:** Author Affiliations: Maureen C. Ashe, Jocelyn M. Chase, University of British Columbia, Vancouver, British Columbia, Canada.

## Abstract

**Introduction:**

Time spent by young adults in moderate to vigorous activity predicts daily caloric expenditure. In contrast, caloric expenditure among older adults is best predicted by time spent in light activity. We examined highly active older adults to examine the biggest contributors to energy expenditure in this population.

**Methods:**

Fifty-four community-dwelling men and women aged 65 years or older (mean, 71.4 y) were enrolled in this cross-sectional observational study. All were members of the Whistler Senior Ski Team, and all met current American guidelines for physical activity. Activity levels (sedentary, light, and moderate to vigorous) were recorded by accelerometers worn continuously for 7 days. Caloric expenditure was measured using accelerometry, galvanic skin response, skin temperature, and heat flux. Significant variables were entered into a stepwise multivariate linear model consisting of activity level, age, and sex.

**Results:**

The average (standard deviation [SD]) daily nonlying sedentary time was 564 (92) minutes (9.4 [1.5] h) per day. The main predictors of higher caloric expenditure were time spent in moderate to vigorous activity (standardized β = 0.42 [SE, 0.08]; *P* < .001) and male sex (standardized β = 1.34 [SE, 0.16]; *P* < .001). A model consisting of only moderate to vigorous physical activity and sex explained 68% of the variation in caloric expenditure. An increase in moderate to vigorous physical activity by 1 minute per day was associated with an additional 16 kcal expended in physical activity.

**Conclusion:**

The relationship between activity intensity and caloric expenditure in athletic seniors is similar to that observed in young adults. Active older adults still spend a substantial proportion of the day engaged in sedentary behaviors.

## Introduction

The amount of time people engage in physical activity tends to decrease with increasing age (1), leading to numerous functional and cardiometabolic sequelae. Older adults make up both the least active and most sedentary cohort in Western countries (2). A lack of energy expenditure from physical activity is considered to be one of the contributors to the growing worldwide rates of obesity (3). Activity profiles of differently aged populations show that the main contributor to calorie expenditure among young adults is time spent in moderate to vigorous physical activity (4), while light activity is the most important contributor to caloric expenditure among older adults (5,6).

Both young adults (7) and older adults (8) spend large quantities of their waking hours engaged in sedentary behaviors, which has cardiometabolic consequences independent of the amount of time spent in leisure-time physical activity. What has not been examined, however, is the activity profiles of older adults who meet guidelines (9) for physical activity (≥150 minutes of physical activity per week). The objective of this study was to measure (by accelerometer) the amount of time active older adults spend in sedentary behavior and to determine which intensity of activity best predicts daily caloric expenditure.

## Methods

This was a cross-sectional observational study. This study was approved by the human subjects committee of the University of British Columbia, and all participants gave written informed consent.

### Participants and recruitment

Fifty-five community-dwelling men and women aged 65 years or older were screened through their affiliation with the Whistler Senior Ski Team of British Columbia, Canada, via a study poster and information session. Participants were enrolled from October 2011 through June 2012.

All participants had to be able to independently perform all basic activities of daily living, climb 1 flight of stairs, and walk 2 blocks without assistance. Current smokers, recreational drug users, and those with diabetes mellitus or cardiovascular disease (prior strokes, transient ischemic attacks, angina, myocardial infarction, or coronary revascularization in the previous 2 years) were not eligible. All participants had to meet current guidelines for physical activity (≥150 minutes of physical activity per week) (9).

### Research procedures

Each participant was required to make at least 1 study visit to allow researchers to collect demographic data and apply an accelerometer. Sensewear Pro armband triaxial accelerometers (BodyMedia, Sword Medical Limited) were fitted snuggly around the left upper triceps and used to monitor levels of physical activity 24 hours per day for 7 full days. Participants were instructed to wear it continuously, including during sleep, except when bathing or swimming. Minute-by-minute epoch data from the SenseWear Pro was analyzed using Body Media InnerView Research Software (version 5.1, BodyMedia, Inc).

To be included in the analysis, participants were required to comply with wearing the accelerometer for at least 5 valid days, including 1 weekend day. A valid day was defined as at least 21 hours of recorded activity on the accelerometer.

### Measures

Accelerometer data was recorded in 1-second epochs. Average time per day spent in sedentary activity, light activity, and moderate to vigorous activity levels was recorded as minutes per day. On the basis of a systematic review of accelerometry practice for older adults (10), the following cut points were used: 99 counts or less per minute as sedentary time, 100 to 1,951 counts per minute as light physical activity, and 1,952 or more counts per minute as moderate to vigorous level of activity (11,12).

The SenseWear Pro armband was also used to measure heat flux (the amount of heat dissipating from the body), galvanic skin response (the amount of evaporative heat loss), and skin temperature (an estimate of the body’s core temperature). These parameters are then entered into proprietary algorithms to estimate caloric expenditure. The use of the SenseWear Pro to measure caloric expenditure due to physical activity has been used in previous investigations in older adults (13) and has been validated against doubly labeled water techniques (14).

### Statistical analysis

All measures of physical activity were normalized by the amount of time per day the accelerometer device was worn. Our primary response variable was caloric expenditure (energy expenditure per day, kcal). The 3 levels of physical activity (sedentary, light, and moderate to vigorous) and predictors in the multivariate linear regression model were determined a priori. Previous investigations showed that age and sex are predictors of energy expenditure in older adults; these predictors therefore were also added to our initial model (11,15).

Scatterplots were visually inspected for outlier data, and density plots were examined to identify data skewing. Any predictors that demonstrated skewing were logarithmically transformed (base 10) before the univariate and multivariate analyses. A tiered approach was used for the analysis whereby the initial model consisted of all of our predictor variables. The data were fitted with a linear model using the least squares method and the parameters (intercept and β coefficients) were calculated (16) as well as scaled β coefficients using standard methods (16). A stepwise method was used to generate each successive regression model; the criterion for removal of variables was the least significant predictor with a *P* value greater than .05. In each iteration of the stepwise regression model, the least significant predictor was removed (17). After the removal of each predictor, an analysis of variance (ANOVA) was performed with the previous model to verify that there had been no significant change. To ensure the assumptions of the multivariate regression were met, variance inflation factors were examined for multicolinearity in each iteration of the model. A value of a variance inflation factor greater than 10 was interpreted as an indicator of collinearity problems (16). Plots of residuals and a QQ plot were examined in our final minimum effective model. The R core software package version 3.0.1 was used for statistical analysis; a significance level of *P* < .05 was set (18).

## Results

Fifty-five people were screened; 1 person was excluded because of a cardiovascular event in the previous 2 years. Of the 54 originally recruited, 2 participants withdrew; 1 participant did not meet the accelerometer compliance criteria, and 1 participant wore the monitor incorrectly. Data from 50 participants (23 men, 27 women) were used in data analysis. The accelerometers were worn for an average of 98.4% (standard deviation [SD], 1.4%) of the study time.

Participants spent an average (SD) of 159 (78) minutes per day in moderate to vigorous activity and 245 (71) minutes per day in light activity (Table 1). Despite this high level of activity, each participant spent a large quantity of time in sedentary behaviors while not lying down (average [SD] = 564 [92] min/d or 9.4 h/d).

**Table 1:** Demographic, Metabolic, and Activity Characteristics of Participants, Study on Physical Activity Among Older Adults (N = 50), British Columbia, 2011–2012

Characteristic	Value
Age, mean (SD) [range], y	71.4 (4.2) [65–81]
Women, n (%)	27 (54)
**Average time in activity level, mean (SD), min/d [% of day^a^]**	
Lying down	480 (57) [33]
Sedentary^b^	564 (92) [39]
Light^c^	245 (71) [15]
Moderate to vigorous^d^	159 (78) [11]

Abbreviation: SD, standard deviation.

a Percentages do not sum to 100% because of rounding.

b Defined as ≤99 counts per minutes as measured by accelerometry.

c Defined as 100 to 1,951 counts per minute as measured by accelerometry.

d Defined as 1,952 or more counts per minute as measured by accelerometry.

None of the predictor variables demonstrated skewing, and no transformation was required before the analysis. In our final minimum effective model, moderate to vigorous physical activity was the only activity parameter significantly correlated with caloric expenditure. Time spent in sedentary activity and light activity was negatively associated with caloric expenditure, but these associations were not significant (Table 2).

**Table 2:** Multivariate Regression Analysis Using Caloric Expenditure (kcal) as Primary Response Variable, Study on Physical Activity Among Older Adults (N = 50), British Columbia, 2011–2012

Predictor	Pearson *R* (95% CI)	*P* Value
**Activity level^a^**		
Sedentary^b^	−0.21 (−0.46 to 0.08)	.15
Light^c^	−0.21 (−0.46 to 0.08)	.15
Moderate to vigorous^d^	0.48 (0.23 to 0.67)	<.001
**Age**	0.01 (−0.28 to 0.29)	.97

Abbreviation: CI, confidence interval.

a All activity levels are in minutes per day.

b Defined as ≤99 counts per minutes as measured by accelerometry.

c Defined as 100 to 1,951 counts per minute as measured by accelerometry.

d Defined as 1,952 or more counts per minute as measured by accelerometry.

A multivariate regression model including 5 predictors (time spent in sedentary activity, time spent in light activity, time spent in moderate to vigorous activity, age, and sex) explained 73% of the variance in caloric expenditure (Model 1, Table 3). The highest variance inflation factor was 2.97 (for time spent in sedentary activity), indicating no issues of multicolinearity.

**Table 3:** Stepwise Multivariate Regression Analysis Using Caloric Expenditure (kcal) as Primary Response Variable, Study on Physical Activity Among Older Adults (N = 50), British Columbia, 2011–2012

Model/Predictors	Unstandardized β (SE)	Standardized β (SE)	*P* Value
**Model 1 **(F_5,44_ = 23.7; *R* ^2^ = 0.73; *P* < .001)			
Time spent in sedentary activity	−7.68 (4.08)	−0.25 (0.14)	.07
Time spent in light activity	15.37 (5.82)	0.33 (0.12)	.01
Time spent in moderate to vigorous activity	24.97 (5.06)	0.64 (0.13)	<.001
Increasing age	−33.92 (61.47)	−0.05 (0.08)	.58
Male sex	4,801.5 (550.0)	1.47 (0.17)	<.001
**Model 2 (**F_4,45_ = 30.0; *R* ^2^ = 0.73; *P* < .001)			
Time spent in sedentary activity	−7.49 (4.04)	−0.25 (0.13)	.07
Time spent in light activity	15.27 (5.77)	0.32 (0.12)	.01
Time spent in moderate to vigorous activity	25.26 (5.00)	0.65 (0.13)	<.001
Male sex	4,725.8 (528.5)	1.45 (0.16)	<.001
**Model 3** (F_3,46_ = 36.9; *R* ^2^ = 0.71; *P* < .001)			
Time spent in light activity	7.58 (4.12)	0.16 (0.09)	.07
Time spent in moderate to vigorous activity	18.14 (3.27)	0.47 (0.08)	<.001
Male sex	4,652.3 (540.8)	1.42 (0.17)	<.001
**Model 4** (F_2,47_ = 51.1; *R* ^2^ = 0.68; *P* < .001)			
Time spent in moderate to vigorous activity	16.28 (3.19)	0.42 (0.08)	<.001
Male sex	4,379.9 (533.1)	1.34 (0.16)	<.001

Abbreviation: SE, standard error.

Model 1 (Table 3) demonstrated positive associations between time spent in light activity, time spent in moderate to vigorous physical activity and male sex with caloric expenditure and a negative association between increasing age and time spent in sedentary activity with caloric expenditure. However, our minimum effective model (Model 4, Table 3), which included only moderate to vigorous physical activity and sex, explained 68% of the variance in caloric expenditure. Every extra minute spent in moderate to vigorous physical activity per day was associated with an increasing caloric expenditure of 16 kcal. In addition, male sex was associated with a higher caloric expenditure (Model 4, Table 3).

One participant had very high levels of activity and was therefore an outlier. A sensitivity analysis in which data for this participant were excluded showed that this exclusion had no effect on the results of the analysis.

## Discussion

We demonstrated that an athletic older adult population spent a substantial portion of their waking hours in sedentary activity (approximately 9.4 hours per day). We also showed that the main contributor to energy expenditure in active older adults is the amount of time spent in moderate to vigorous activity.

The most surprising finding of our study was that despite exceeding the current guidelines for physical activity of 150 minutes or more of physical activity per week (9), highly active older adults spent a large quantity of the day completely sedentary. The amount of sedentary time observed in our active population was comparable to that seen in sedentary adults over 60 years old (19), sedentary adults over 65 years old (6), middle-aged sedentary adults (11), adult men over 70 years old (20), and older adult men over 80 years old (20). This amount of sedentary time was surprising given that the physical activity level of our participants is observed in less than 5% of the older adult population (21).

Although the contribution of different levels of activity to energy expenditure has been studied in sedentary young (4) and sedentary older (6) adults, this relationship has not been examined in active older adults. Previous work showed that the main contributor to energy expenditure in young adults is moderate-to-vigorous intensity activity (4). In contrast, light activity has been shown to be the main contributor to energy expenditure in older adults (5,6). Our participants demonstrated a relationship between activity intensity and caloric expenditure that was more in keeping with a younger population, with moderate-intensity activity predicting energy expenditure.

Although the reason for this “young” profile for activity and energy expenditure is unclear, some exercise intervention studies suggest an underlying mechanism. Unlike young adults, older adults may increase their physical activity though a shift from sedentary to light intensity activities, because these activities tend to be better tolerated (22). Our participants clearly did not follow this pattern, perhaps because most of our participants were continuing an established pattern of high levels of physical activity as opposed to starting an exercise program from a previous sedentary state.

Although regular leisure-time physical activity has many benefits (23,24), sedentary behavior has recently been identified as an independent risk factor for dyslipidemia, diabetes mellitus, obesity, and hypertension (23,25). Of even more concern, these associations persist despite accounting for the level of moderate and vigorous physical exercise (26). These findings suggest that sedentary behavior may pose a risk for cardiometabolic disease that is distinct from physical exercise, or the lack thereof. Our study population was an extremely active group of individuals; despite this, they spent a large amount of time in sedentary behaviors. In fact, the time spent sedentary was similar to that observed in studies of average normal older adults (6). Although more work needs to be done, our results suggest that even active older adults could benefit from interventions (such as the use of standing desks) that would reduce sedentary time without interfering with their current high levels of moderate-intensity activity.

Our study has several potential limitations. The cross-sectional nature of the study design limits inference about causality. Prospective or interventional trials are needed to define the physiologic and behavioral factors involved in the associations observed in this study. In addition, our highly active study population and the study’s small sample size make generalizability of our results to less active populations problematic.
